# Levelling the playing field: students’ motivations to contribute to an amnesty of assessment materials

**DOI:** 10.1186/s12909-020-02320-0

**Published:** 2020-11-23

**Authors:** Anjali R Gondhalekar, Eliot L Rees, Daniel Ntuiabane, Osman Janjua, George Choa, Oziegbe Eboreime, Alison Sturrock

**Affiliations:** University College London Medical School, 74 Huntley Street, London, WC1E 6JE England

**Keywords:** Cheating, Inequity, Fairness, Qualitative, Focus group, Medical education, Undergraduate, Examination

## Abstract

**Background:**

‘Exam recall’ is a recognised phenomenon whereby students recall and record questions after leaving the examination hall. This poses two main problems. First, as these questions are only available to peers of the students who recall the questions, these individuals have an unfair advantage. Secondly, the distribution of these recalled questions poses a threat to the validity and defensibility of assessments.

To address the first of these problems, we developed an amnesty enabling students to submit assessment material to an on-line site. This study sought to explore which factors influence students’ contributions to an amnesty of assessment material.

**Methods:**

We conducted a qualitative study using semi-structured focus groups. We used convenience sampling and recruited participants from all years of our undergraduate medical programme. The focus groups were facilitated by a medical student peer to reduce the power imbalance and encourage participants to discuss candidly. The focus groups were audio recorded and transcribed verbatim.

Two researchers independently analysed all transcripts using thematic analysis and the research team met regularly to discuss emergent findings. Nvivo was used to assist with thematic analysis of the transcripts.

**Results:**

Twenty-six individuals participated in six focus groups. Six themes were identified through the analysis, which were categorised into motivating factors and de-motivating factors.

Motivating factors were a perception that this would overcome inequity, a fear of repercussions, and the perceived usefulness of resources. Factors that prevented students contributing were a culture of competition, a lack of incentives, and mistrust of the medical school.

**Conclusions:**

The establishment of an amnesty was acceptable to students and they were motivated to contribute materials. The competitive nature of medical careers and the stakes of summative assessments meant that students felt that some peers might still not contribute their materials. Students felt that the school were listening to their concerns and this led to a better dialogue between students and faculty.

## Background

Cheating is rife in medical education. One study estimates that up to 58% of medical students have cheated [1]. This is a serious problem for three key reasons. Firstly, if students cheat on assessments then medical schools’ judgements on competence may be flawed. Secondly, cheating is dishonest and unprofessional behaviour. It has been demonstrated that unprofessional behaviour in medical school is associated with future deficiencies in professionalism, and subsequent disciplinary action [2, 3]. Finally, when performance in medical school is norm referenced (i.e. when applying for postgraduate training jobs), cheating puts some individuals at an unfair advantage.

There are many ways in which students cheat in assessments [4]. One such way is ‘examination recall’, where students attempt to remember the questions that were in an assessment to pass them on to others [5]. One study found that as many as 89% of students participate in exam recall [6].

This high prevalence of cheating may seem surprising. However, many students engaged in these practices would not consider their activities to be cheating. Indeed, depending on how one defines ‘recall’, as few as 18% of students perceive these behaviours as unethical [7].

In many contexts, sharing previous assessment items has become part of the culture among medical students. Students may feel obliged to engage in exam recall as it is seen as ‘the norm’ [8]. Nevertheless, this behaviour is a key area of concern for regulators. For example, in the UK context the General Medical Council would consider this misconduct as evidence of impaired fitness to practice [9].

Many programmes reuse assessment items to limit the burden of creating new items [10]. Students sharing questions recalled from assessments, therefore, are likely to negatively influence the psychometric properties of compromised material and jeopardise the integrity of future examinations [11]. Whilst there is limited knowledge of the implications of sharing items on the integrity of examinations, Wagner-Menghin et al. concluded that there was little impact of question exposure in the context of a formative assessment but was unable to observe statistical differences to reflect this [12].

### Context

In the UK it is common for university students to be members of student societies and sports clubs, including groups specifically for medical students. In the academic year of 2018/19, concerns were raised by students at University College London Medical School (UCLMS) regarding members of student societies and sports clubs acquiring assessment material through examination recall and sharing them amongst these groups. In response, UCLMS introduced an assessment amnesty whereby medical students of all year groups were invited to submit (anonymously if preferred) assessment material they had in their possession. As a result of this amnesty, any student who was found in possession of material that had not been submitted would be perceived by the medical school as actively withholding assessment material. Students in this position would then be referred to Fitness to Practise procedures [9].

This study sought to explore which factors and characterise the factors that influence students’ contributions to an amnesty of assessment material.

## Methods

This study was conducted at University College London Medical School (UCLMS). Ethical approval was granted from University College London’s Research Ethics Committee (VPRO ID: 12725/003).

### Design

As there has been no previous research regarding assessment amnesties in medical education, we conducted an exploratory qualitative focus group study taking a constructivist perspective [13]. Constructivist research is collaboratively constructed through dialogue between researchers and participants and findings are interpreted by the researchers [14]. As such, the backgrounds, beliefs and values that the researchers bring to the study are of significance. We established a research team comprised of medical students (DN, GC & OE) and clinical academics with roles in teaching (AG & OJ) and assessment (ER & AS). This helped us to interpret the data through different perspectives.

### Participants

We utilised a convenience sampling approach in which students from all six years of the undergraduate medical programme were invited to participate. Students received an invite to participate in semi-structured focus groups discussing assessment and feedback at UCLMS via email. Interested students were instructed to contact the student representative conducting the research. Students were not asked to specify whether they were members of any sports clubs or societies. Participants were provided pizza and refreshments. No other incentives were offered. In total, 26 students participated in the study and attended the focus groups.

### Data collection

Focus groups were chosen as they offered an opportunity to explore contrasting views amongst medical student peers [15]. Semi-structured focus groups were conducted loosely following a topic guide which was constructed through discussion between staff and student researchers. The focus groups were held in the medical school building and were facilitated by a medical student peer (DN, OE or GC) to reduce any perceived power imbalances and encourage participants to discuss the amnesty with candour and without fear of repercussions [16, 17]. All student facilitators were trained in qualitative data collection methods by faculty members. Faculty members were otherwise not involved in data collection. One focus group was held for each year group of the medical school.

The focus groups were audio recorded and transcribed verbatim by an independent transcription company. The transcripts were reviewed for accuracy and anonymised.

### Data analysis

We employed thematic analysis as described by Braun and Clarke [18]. The research team independently read and familiarised themselves with all of the transcripts. Two researchers (AG & ER) analysed the first two focus groups coding inductively from the data. They met to discuss and confirm the identified codes and then both independently analysed all transcripts. The research team met regularly throughout analysis to discuss the codes and develop thematic categories. After all transcripts had been analysed, two researchers reread each transcript and coded the data to each theme to review fit and coverage [19]. The research team then met again to discuss emergent findings.

In order to maintain reflexivity throughout the research process, during each meeting we discussed amongst the group how our perspectives might be influenced by our roles within teaching and assessment.

Analysis was conducted using NVivo 12 (QSR).

## Results

**Fig. 1:**
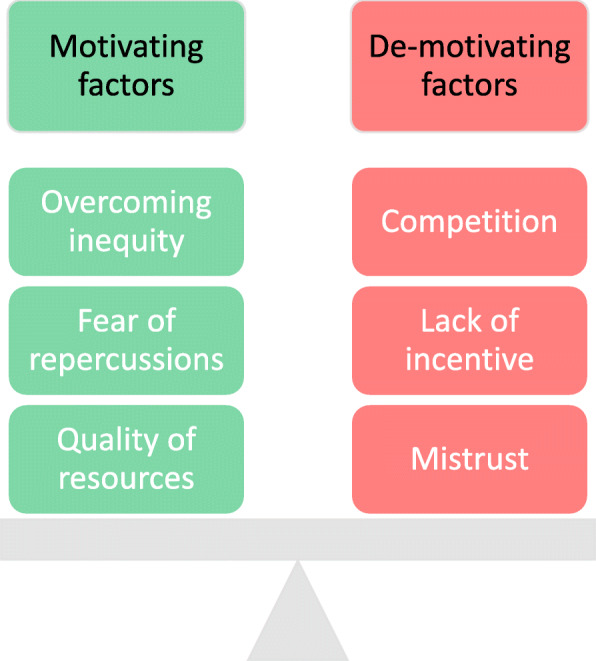
Themes identified from thematic analysis

Twenty-six individuals participated in six focus groups lasting a mean of 48 min (range 28–63). Six themes were identified through the analysis, which are categorised into motivating factors (those that encouraged students to submit materials to the assessment amnesty) and de-motivating factors (those which discouraged students from submitting materials) (Fig. 1). We will present each theme in turn with indicative quotations.

### Motivating factors

Participants welcomed the introduction of the assessment amnesty and described it as a useful resource for learning and for preparing for summative assessments. They described three main reasons for why students may contribute assessment materials: overcoming inequity, fear of repercussions, and the usefulness of the resources.

#### Overcoming inequity

Participants describe a sense of inequity in the medical school, whereby there was a perception that certain social groups had access to past assessment materials and others did not.

*“I think my understanding was that it was … that some students had access to more information that was useful in exams than others because of like you know being in [team sports] as an example” (FG2)*

*“I don’t see why if some people have access to question banks that other people shouldn’t. I mean just from that very basic perspective.” (FG1)*

They felt that the introduction of an assessment material amnesty ‘levelled the playing field’ and helped move towards overcoming this inequity.

*“I kind of see it as like the analogy of like legalising a drug or something that you’re regulating it, you’re taking control, so that there’s not this like black market of questions going round to the people that are lucky enough to have access to it.” (FG2)*

*“The premise is good, you know standardising the same content available to everyone, rather than you know certain people within certain groups, such as societies, only having access to the best material potentially.” (FG4)*

#### Fear of repercussions

Participants described concern amongst the student body about contributing to the amnesty for fear of getting in trouble with the medical school.

*“Everyone’s been emailing like oh you’re going to get in trouble if you don’t submit your materials and everything” (FG5)*

*“I think people were apprehensive about like getting into trouble, and I know it was made really clear that it was an amnesty and that you know no one is really going to get in trouble, but people were like why do I have to submit this … ” (FG4)*

#### Quality of resources

Participants described feeling there was a lack of assessment-related resources for them to use when preparing for summative assessments.

*“And I think the fact that there are so many Google Drives and SBA banks and stuff just shows how desperate people are for those mock questions and the practice that you really need to prepare yourself for an exam and there’s just not enough provided by the medical school, so you have to go to the black market.” (FG2)*

They felt that sharing the assessment materials that were currently on the ‘black market’ would also enable the medical school to feed back on their utility as learning resources.

*"I was reading the PDF that was up on Moodle about this, and it said like the materials that have been handed in are like unhelpful and actually not what UCL want you to learn - and that’s important for us to know as well. I think if you also … as part of the framing in a positive light would say’If you give us this we can give you feedback on what’s useful and what’s not, and we can upload the stuff that’s useful and you can download it, and this big OSCE pack that’s been going round for years and years that’s awful, don’t go near it because it’s not going to help you prepare for the real OSCE’ - and whatever, like that feedback is useful to us when we’re studying" (FG1)*

### Withholding assessment materials

Participants, however, were cautious that not all materials had been submitted. They described three main reasons for why students might not contribute materials to the assessment amnesty: competition, lack of incentive, and mistrust.

#### Competition

Firstly, they recognise the importance of their performance relative to their peers. In the United Kingdom, medical students’ rankings (in terms of deciles) contribute significantly to their scores on applications for medical graduates’ first postgraduate jobs (the foundation programme). Consequently, there was a perception that students wanted not only to perform well on assessments, but to perform better than their peers. This resulted in a culture of competition, which may lead to students choosing to withhold materials from the amnesty to preferentially advantage themselves and their friends.

*“Just that people don’t want to necessarily share what they’ve got … ” (FG2)*

*“But some people don’t want that. If everybody wanted a level playing field then everybody would share their resources.” (FG6)*

*“If people are giving in SBA materials that they shouldn’t have … they’re no longer useful [ … ] the reason people are using them is so that they know previous questions – it gives them the upper hand.” (FG1)*

In final year, when their summative assessment scores do not contribute to their postgraduate applications, they describe a more collaborative culture.

*“I think that’s an atmosphere of final year, everyone wants to help each other, because there’s no deciles or anything” (FG1)*

*“It’s got a lot more collaborative, and it’s got a lot more … everyone wants everyone to succeed, … ” (FG1)*

#### Lack of incentive

Participants described a lack of incentive for students to contribute their materials to the amnesty.

*“And the problem is how … you know there are still going to be groups of people [ … ] who have you know the best resources, and what incentive do they have to give it?” (FG6)*

*“And why would a person in that year give it in when they know that if they keep hold of it it could potentially come up in an exam?” (FG6)*

As they felt that not everybody would contribute their materials, they felt this could potentially disadvantage those that do relative to those that do not.

#### Mistrust

Participants were also cautious about the motivations of the medical school in creating the assessment material amnesty. Some were sceptical the resources would be shared amongst the students.

*“Not meaning to be sceptical here, I’d be really excited to see the med school actually helping us by using our resources for us and not just being like ‘Well we just want to take it’ – take it away” (FG3)*

*“I don’t know, say people handed in like questions or whatever – will we actually see those questions?” (FG3)*

## Discussion

This study sought to explore the factors that motivate students to contribute to an amnesty of assessment materials. We found a strong sense of collaboration amongst students wanting to improve the equity of access to assessment resources for everyone. Some students contributed to the amnesty out of fear of repercussions from the medical school or General Medical Council (GMC) for not submitting assessment materials in their possession. This suggests that the introduction of the amnesty raised awareness amongst students that distributing assessment materials obtained illicitly (e.g. through exam recall) was unprofessional behaviour.

Whilst many students described a desire to ‘level the playing field’ in terms of access to assessment materials and have supported the ethos of the amnesty, some students were sceptical about whether all students are likely to contribute to the amnesty. This is perhaps due to the perceived advantage that some students have by withholding assessment resources in enabling them to achieve overall higher educational performance relative to their peers.

In the UK, students’ first postgraduate training job applications are allocated based on a mix of overall performance at medical school, and performance on a situational judgement test [20]. The high stakes nature of these job applications means that students not only want to perform well in their assessments in order to pass, but they want to perform better than their immediate peers in order to be ranked more highly. This external pressure is likely to influence behaviour. This behaviour may not be limited to learning [21].

This is the first study exploring students’ engagement with an innovation aimed at addressing cheating culture in a medical school and has identified some novel insights. Our study benefits from the involvement of students in the recruitment, organisation, and facilitation of the focus groups and their contribution to the data analysis.

These strengths notwithstanding, this study has a number of limitations.

Firstly, it was undertaken at a single medical school. While these findings may be transferable to other medical schools who adopt an amnesty approach, the culture within the school is likely to influence students’ engagement.

Secondly, the focus groups were undertaken prior to the release of amnesty assessment material. Consequently, students were unable to comment during focus groups on the quality of the submitted assessment material, its impact on their exam preparation, and usefulness as a revision resource.

Finally, we used a convenience sampling approach. Participants were a self-selecting group, who may not be representative of the wider student body in each year group. However, we did find that the major themes identified across the different focus groups (with different year groups) were consistent.

This study provides insight into students’ perceptions of the introduction of an assessment amnesty. We are yet to see the full impact of the introduction of the assessment amnesty in terms of how medical students value the amnesty material, how it has been utilised, and whether students will continue to recall and share new assessment items. Furthermore, we do not yet know what the effect of releasing these assessment items in the form of an amnesty will have on the psychometric properties of the items.

Amidst speculation about the equality that the assessment potentially offers, there is still a great deal of debate on whether the amnesty can ever truly level the playing field. Moreover, whilst there is some suggestion that the student body is more likely to trust the motives of the medical school, further data into students’ reservations about the medical school are yet to be explored.

Furthermore, we need to consider the implications for those students that do not submit assessment material in their possession and how action taken against such individuals could further impact on the relationship between faculty and students. Moreover, it is clear that there is a fine balancing act to be maintained between the need to develop a culture of trust between faculty and student whilst respecting the boundaries that have been outlined by the institution, which remains accountable for it assessment processes.

Future research should focus on psychometric analyses of items in the assessment bank that are known to be available to students' (e.g. in an amnesty), students use of the amnesty resources, and the effect of an amnesty on the culture of cheating.

## Abbreviations

UCLMS: University College London Medical School
DN: Daniel Ntuiabane
GC: George Choa
OE: Oziegbe Eboreime
AG: Anjali Gondhalekar
OJ: Osman Janjua
ER: Eliot Rees
AS: Alison Sturrock
QSR: QSR International
FG: Focus group
VPRO: Vice Provost (Research) Office
GMC: General Medical Council
